# Platelets in Renal Disease

**DOI:** 10.3390/ijms241914724

**Published:** 2023-09-29

**Authors:** Drolma Gomchok, Ri-Li Ge, Tana Wuren

**Affiliations:** 1 Research Center for High Altitude Medicine, School of Medicine, Qinghai University, Xining 810001, China; gomchokdolma@163.com (D.G.); geriligao@hotmail.com (R.-L.G.); 2 Key Laboratory for Application for High Altitude Medicine, Qinghai University, Xining 810001, China

**Keywords:** platelets, renal disease, hemostasis, inflammation, immunity

## Abstract

Kidney disease is a major global health concern, affecting millions of people. Nephrologists have shown interest in platelets because of coagulation disorders caused by renal diseases. With a better understanding of platelets, it has been found that these anucleate and abundant blood cells not only play a role in hemostasis, but also have important functions in inflammation and immunity. Platelets are not only affected by kidney disease, but may also contribute to kidney disease progression by mediating inflammation and immune effects. This review summarizes the current evidence regarding platelet abnormalities in renal disease, and the multiple effects of platelets on kidney disease progression. The relationship between platelets and kidney disease is still being explored, and further research can provide mechanistic insights into the relationship between thrombosis, bleeding, and inflammation related to kidney disease, and elucidate targeted therapies for patients with kidney disease.

## 1. Introduction

Acute kidney injury (AKI) and chronic kidney disease (CKD) are two different types of kidney disease that can be caused by various etiologies, including medicines and toxins, ischemia, infections, hypertension, diabetes, and autoimmune illnesses. AKI occurs in approximately 10–15% of hospitalized patients, while its incidence in intensive care has been reported in more than 50% of patients [1]. Furthermore, it is concerning that the number of patients affected by CKD has been increasing, affecting >10% of the general population worldwide [2]. All types of renal injury can develop into severe end-stage renal disease (ESRD), for which renal replacement therapy is the only curative option [3,4]. Moreover, AKI and CKD are interrelated and may function as risk factors for one another. These conditions are typically related to an elevated risk of cardiovascular incidences and morbidity and mortality rates [1,5]. Despite extensive research, treatments for inhibiting the progression of renal disease and its related complications still face significant challenges.

The kidney receives 20–25% of the cardiac output blood through a comprehensive vascular network [6] to ensure its important roles in metabolism, excretion, and endocrine function. The kidney’s endothelial, epithelial, interstitial, and immune cells orchestrate and maintain homeostasis in the body [6,7,8]. During kidney disease, the structure and function of the kidneys are damaged, and homeostasis deteriorates. More importantly, it can cause inflammation and exacerbate pathological and physiological processes, including oxidative stress, acidosis, ischemia, and hypoxia, further promoting kidney disease progression and causing multiple organ and tissue lesions [7,9]. Since Morgagni first described the association between bleeding and renal dysfunction [10], increasing evidence has confirmed the close relationship between blood homeostasis and kidney disease.

Platelets are prevalent in blood and are essential for maintaining vascular integrity and hemostasis. In addition to their well-known function in hemostasis, platelets have recently gained increasing attention for their essential functions in controlling inflammation and immunity [11,12]. According to clinical observations, patients with kidney disease frequently experience platelet abnormalities, and growing evidence indicates a bidirectional relationship between platelets and renal diseases; kidney disease can cause platelet abnormalities, and vice versa.

Since kidney disease poses such a serious threat to public health, efforts are being made to improve current treatment options. Further understanding of the mechanisms of platelet function in renal diseases may provide potential insights into the rational treatment of kidney disease and its complications. However, the exact relationship between platelets and kidney disease remains unclear, making it challenging to determine how to administer antiplatelet therapy to patients with kidney disease without increasing the risk of bleeding. Moreover, the antiplatelet drugs commonly used in clinical practice are systemic agents, and when they are used to prevent kidney disease progression, the risk of bleeding cannot be predicted.

In this review, we focus primarily on the interaction mechanisms between altered platelets and renal diseases to provide insights into managing platelets in kidney disease and the development of therapies targeting platelets to prevent kidney disease progression.

## 2. Platelet Biology

Platelets are the blood cells present in the highest number after erythrocytes (150–400 billion cells/L in the human blood). Platelets are small (2–4 μm) anucleate cells derived from megakaryocytes [12]. They patrol the blood circulation with a stable disk shape and can rapidly react to any biological changes in the vessel wall caused by injury or infection. Despite lacking genomic DNA, human platelets retain up to 45% of reference sequence database (RefSeq) genes as messenger ribonucleic acid (RNA) (mRNA). Remarkably, platelet mRNAs can support de novo protein synthesis [13]. Moreover, they contain various surface receptors, adhesion molecules, and abundant granules [14,15,16]. Table 1 provides an overview of the major components stored in platelet granules. Once activated, platelets begin to change shape and surface molecules, degranulate, and release organelles and microvesicles or molecules in response to different stimuli [14,17,18].

**Figure 1:**
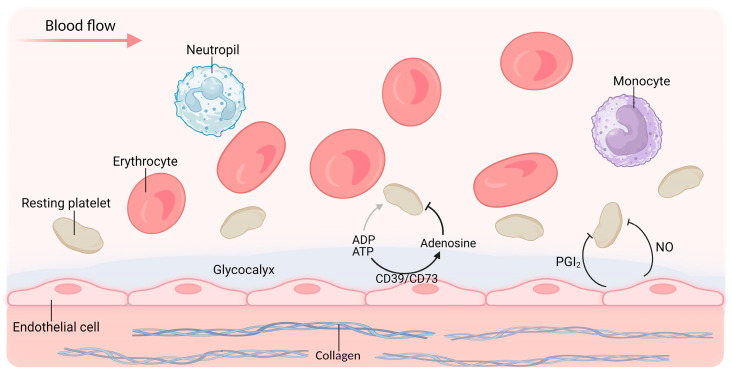
Platelets circulate in a resting state under physiological conditions. In the bloodstream, platelets do not interact with leukocytes, and erythrocytes increase platelet–vessel wall contact by displacing platelets away from the axial flow and toward the vessel wall, which allows platelets to respond quickly to potential vascular damage. The intact glycocalyx and endothelial cells lining the vessel wall prevent platelet adhesion and subsequent activation. The healthy endothelium inhibits platelet activity by releasing nitric oxide (NO) and the eicosanoid prostacyclin (PGI2). Furthermore, the expression of ectonucleases CD39 and CD73 on the endothelium hydrolyzes ATP and ADP into the platelet inhibitor adenosine. Created with BioRender.com.

**Figure 2:**
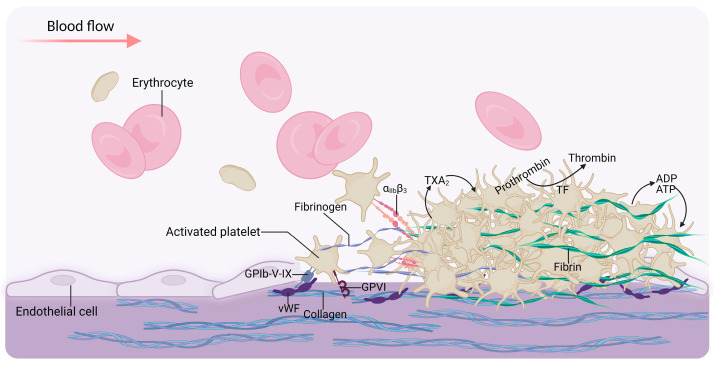
Platelets in thrombus formation. The damaged endothelium exposes the subendothelial matrix proteins von Willebrand factor (vWF) and collagen. Platelet adhesion to vWF (via GPIb–V–IX) and collagen (via glycoprotein VI (GPVI) receptors) trigger thrombus formation. Fibrinogen binding to activated integrin α_IIb_β_3_ then triggers platelet aggregation. Activated platelets simultaneously release mediators, including ADP and thromboxane A_2_ (TXA_2_), attracting circulating platelets to the growing thrombus. Additionally, the tissue factor (TF) exposed by the injured vessel wall induces the production of thrombin, which converts fibrinogen to fibrin, thereby forming a stable, fibrin-rich clot. Created with BioRender.com.

**Figure 3:**
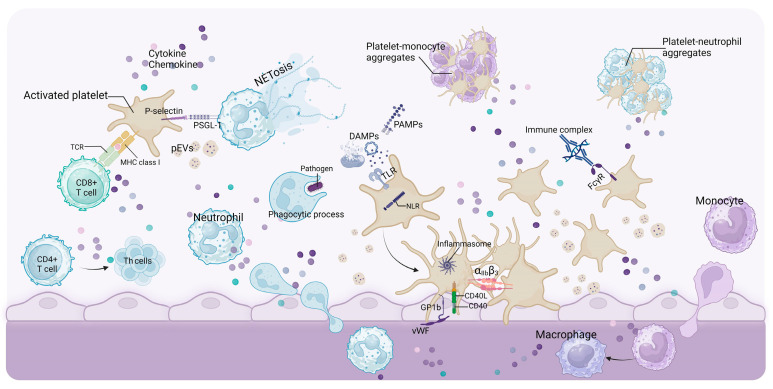
Platelets in inflammation and immunity. Platelets contribute to inflammation and immune responses via various pathways. Activated platelets release various chemokines and cytokines, promoting leukocyte recruitment, infiltration, differentiation, phagocytosis, cytokine release, and boosting inflammation and immune function. P-selectin on activated platelets binds to P-selectin glycoprotein ligand 1 (PSGL-1) on leukocytes to form platelet–neutrophil and platelet–monocytes heterotypic aggregates and stimulate the formation of neutrophil extracellular traps (NETs), playing an essential role in host defense. DAMPs and PAMPs in the environment combine with PRRs, such as TLRs on platelets, enhancing the innate immune effect and promoting the activation and aggregation of platelets. Furthermore, activating NLRP3 causes pyroptosis and forms inflammasomes, exacerbating the inflammatory response. The cytokines derived from platelets facilitate Th cell differentiation. Moreover, platelets express major histocompatibility complex (MHC) class I, which can present antigens to CD8^+^ T cells. Additionally, immune complexes can stimulate platelet activation in a FcγR-dependent manner. In summary, platelets exert powerful inflammatory and immune effects, and are interrelated with hemostasis. Created with BioRender.com.

The primary function of platelets is hemostasis [20]. Under physiological conditions, platelets are in a resting state (Figure 1). However, upon vascular injury, the subendothelial surface (the primary site of platelet activation) can be quickly anchored by platelets. They form plugs (primary hemostasis), which are subsequently reinforced with fibrin strands via a coagulation cascade (secondary hemostasis) [21,22,23] (Figure 2). In addition to their hemostatic effect, platelets play crucial roles in both inflammatory and immune responses. Once activated, they can interact with leukocytes, thereby stimulating mutual activation and leading to the rapid and localized release of platelet-derived cytokines. This mechanism enables platelets to modulate leukocyte effector functions and contribute substantially to the inflammatory and immune responses triggered by injury or infection [24,25,26] (Figure 3). Hemostasis, inflammation, and the immune system of platelets coordinate to provide vital protection against injury and foreign invaders. Therefore, dysregulation of these responses will have pathological consequences for the host.

## 3. The Mechanisms of Platelet Abnormalities in Kidney Disease

Owing to the kidney’s vital role in homeostasis, renal lesions are not limited to local areas of the kidneys, but also seriously affect the internal milieu. AKI is characterized by a rapid decline in renal function, whereas CKD is associated with a gradual loss of renal function over time. Nevertheless, both conditions are accompanied by almost the same pathophysiological changes, including fluid overload, acidosis, electrolyte abnormalities, and blood component abnormalities (e.g., anemia and uremia). Moreover, CKD progression is accompanied by multiple complications, such as cardiovascular disease, high blood pressure, and bone loss. Therefore, in this milieu, platelets in the blood circulation are vulnerable to being affected. Hence, it is crucial to understand the changes in platelet function in renal disease, because patients with AKI or CKD may possess different hemostatic states, even at the same stage. Moreover, both bleeding and thrombosis, which are contradictory outcomes, can occur in the same patient, particularly in those with ESRD, and this paradoxical outcome directly affects clinical decision making. Although platelet abnormalities in kidney disease have attracted the attention of clinicians and researchers, the mechanism underlying these abnormalities is extremely complex, and requires further research.

### 3.1. Abnormalities in Antiplatelet Factors

The kidney has a wide distribution of endothelium in the arterioles, glomerular capillaries, and post-glomerular capillary network which encircles the tubules. In various renal pathologies, progressive impairment triggers increased endothelial activation and inflammation, reduced endothelial proliferation, and enhanced senescence, ultimately leading to diminished endothelial integrity and increased permeability [27,28,29,30]. Endothelial injury contributes to reducing antiplatelet mediators (glycocalyx, NO, PGI2, CD39/CD73), which triggers platelet activation and aggregation [31].

#### 3.1.1. Glycocalyx

As previously mentioned, an intact glycocalyx is vital for maintaining vascular integrity. One reason is that it can act as a negatively charged molecular sieve to limit the transvascular movement of negatively charged and/or large molecules [32,33,34]. Notably, the glycocalyx covering the endothelial cells of the glomerulus is a key component of the glomerular filtration barrier [7]. Moreover, the glycocalyx can limit the interaction between leukocytes and the endothelium through endothelial cell-associated adhesion molecules, including integrins and immunoglobulin superfamilies. Furthermore, it can protect endothelial cells from oxidative stress and sense fluid shear force, transmitting it to the endothelium and triggering NO production, thereby inducing vasodilation and reducing the adhesion of white blood cells and platelets [31]. Various clinical conditions, including AKI [35,36], diabetic kidney disease (DKD) [37,38], and CKD [39,40,41] due to other causes, can damage the endothelium and loss of glycocalyx.

Under AKI induced by ischemia-reperfusion (I/R) or sepsis, glycocalyx deterioration was observed, which induced endothelial activation, thereby promoting the activation of platelets and their interaction with the endothelium [42,43,44,45]. Studies on animals have demonstrated that inhibiting glycocalyx degradation can lessen platelet adhesion after an I/R injury, and alleviates acute tissue injury [45,46,47]. Therefore, we can reasonably infer that glycocalyx disruption, the first line of endothelial defense, may significantly impact platelet abnormalities in renal diseases.

#### 3.1.2. NO

Nitric oxide (NO), the smallest known signal molecule, is produced from L-arginine by NO synthase (NOS) [48]. NO is a ubiquitous intra- and extracellular messenger implicated in diverse signaling pathways that affect various physiological processes, including ion channel modulation, gene transcription, inflammation, immunity, and vascular tone [48,49]. By boosting the production of cyclic guanosine monophosphate, NO reduces platelet–platelet interactions [50,51]. Dysfunction of the endothelial cells can reduce NO synthesis [52,53,54], which may influence platelet reactivity [55]. Importantly, NO is involved in various physiological and pathological processes in the kidney [56,57]; therefore, changes in NO levels may affect the state of platelets in kidney diseases.

A significant decrease in epithelial NOS has been observed in AKI models [58,59]. Other studies have suggested a positive correlation between endothelial NOS levels and the severity of AKI [60,61]. The above results are not only related to the regulation of vasodilation by NO, but may also be associated with the inappropriate activation of platelets caused by a reduction in NO levels [62]. Similar changes have been observed in diabetes [63]. Moreover, multiple clinical studies have demonstrated decreased platelet aggregation in patients with diabetes receiving intravenous L-arginine [63,64,65]. This finding supports the hypothesis that endothelial damage accompanied by decreased NO synthesis leads to inappropriate platelet activation.

Intriguingly, not all cases of kidney disease are accompanied by decreased NO levels. Patients with uremia may have increased plasma NO metabolite levels [66,67,68], enhanced platelet NO generation [69,70,71], and elevated NO levels in exhaled air [72]. In vitro, plasma from patients undergoing hemodialysis significantly stimulates NO synthesis in human umbilical vein endothelial cells [69]. The stimulatory function is explained by elevated levels of cytokines, including tumor necrosis factor-α and interleukin (IL)-1b (which are effective inducers of the inducible isoform of NOS) in patients with chronic renal failure receiving or not receiving maintenance hemodialysis [70,73,74]. However, for ESRD, there is still controversy in the literature regarding changes in NO levels, with most studies suggesting their increase and some reporting a decrease [75,76]. This may be attributed to the patient’s blood pressure, dialyzer response, clearance of endogenous NOS inhibitors via dialysis, and heparin administration [66,77].

#### 3.1.3. PGI2

Prostacyclin (PGI2) is a metabolic product of arachidonic acid released by membrane phospholipids under the action of cyclooxygenase and PGI synthase (PGIS), which are predominantly produced by endothelial cells [78]. PGIS has been identified in the mesangial cells, interstitial cells, and peritubular capillaries of human kidneys [79]. PGI2 has been demonstrated to suppress vasoconstriction and inhibit platelet aggregation by increasing cyclic adenosine monophosphate levels in platelets [80]. Multiple investigations have revealed that PGI2 dysfunction is associated with the development of various cardiovascular diseases [81] and their pathogenesis, including thrombosis and circulatory disorders [82]. For example, Yokoyama et al. showed that PGIS-deficient mice had nephrosclerosis and renal infarction [83]. However, renal-specific overexpression of PGIS in mice, which increases PGI2 levels, can prevent endotoxemia-induced AKI [84]. Notably, similar to NO, endothelium-derived PGI2 levels may increase rather than decrease in uremia [85,86,87]. Consistent with this, in vitro studies have shown that plasma from patients with uremia produces more PGI2 than endothelial cells stimulated by normal plasma [88].

#### 3.1.4. CD39/CD73

CD39 and CD73 primarily regulate the balance in the blood and extracellular fluid between extracellular ADP/ATP and adenosine, which inhibits platelet adhesion and aggregation [89]. Recently, many studies have indicated that this axis plays a vital part in I/R injury, renal fibrosis, diabetic nephropathy, transplantation, hypertension, and inflammation, among others [90,91].

CD39 is the main producer of adenosine in I/R injury that occurs in the kidney, liver, bowel, heart, lung, brain, and islet cells [92]. However, in an in vitro model of oxidative stress and inflammation in the aorta of humans and pigs, a decrease in CD39 promoted ADP buildup, further enhancing platelet aggregation [93]. In I/R models, such as in ischemia in the heart, kidneys, and liver, CD39 downregulation is common, and the absence of CD39 implies yet more severe damage [94,95,96,97]. Correspondingly, transgenic mice overexpressing CD39 were protected from warm and cold I/R injury (IRI) in the kidneys [98]. Similarly, administering apyrase, a soluble form of CD39, raised adenosine levels in tissues, thereby eliminating IRI in the kidneys [97]. Therefore, despite the lack of direct evidence, it is reasonable to assume that the protective effect of CD39 is likely related to the inhibition of platelet aggregation and improvement of microcirculation, in addition to improving inflammation and immune status. Moreover, higher levels of CD73 expression have been found in kidney biopsies from patients with CKD than in controls [99]. Currently, hypertension and diabetic nephropathy are still the main causes of CKD. It has been reported that changes in platelet activity in patients with diabetes and hypertension are associated with increased expression of CD39 [100,101].

Overall, the primary task in preventing platelet abnormalities in patients with kidney disease is to avoid widespread endothelial injury and disruption of the internal environment. Due to the direct relationship between antiplatelet factors and endothelial integrity, once platelets are activated, the subsequent cascading reactions of coagulation and inflammation form a positive feedback loop. Therefore, correcting the inflammatory state and maintaining a stable internal environment are crucial to prevent aberrant platelet activation in patients with kidney disease.

### 3.2. Abnormalities in Platelet-Activating Mediators

In kidney disease, damage to the renal endothelium leads to an imbalance in platelet-activating factors (TF, thrombin). Moreover, some special causes, such as sepsis-induced AKI, directly cause kidney and platelet abnormalities due to lipopolysaccharides (LPS) acting as pathogen-associated molecular patterns (PAMPs). Furthermore, with the loss of renal function, abnormal components, including damage-associated molecular patterns (DAMPs), uremic toxins, excessive reactive oxygen species (ROS), and other substances, may accumulate, leading to platelet abnormalities.

#### 3.2.1. TF

Tissue factor (TF) is expressed in perivascular and epithelial cells in various organs and tissues, where it forms a hemostatic barrier. Under normal circumstances, TF does not exist in the circulation. TF is only exposed to circulation when the integrity of the vascular wall is disrupted and exerts a hemostatic effect by activating the coagulation cascade reaction [102]. As previously mentioned, TF initiates thrombin formation and subsequently activates platelets. Fibrinogen and fibrin can activate platelets by binding to either integrin α_IIb_β_3_ or glycoprotein (GP)VI.

Increased TF levels were found in the kidneys of AKI models induced by I/R and sepsis [103,104]. In another primate model of sepsis, TF inhibition reduced renal fibrin deposition and preserved renal function [105]. Importantly, under CKD conditions, uremic toxins, including indoxyl sulfate (IS) and indole-3 acetic acid, directly increased the prothrombotic activity of the endothelium, as reflected by increased TF expression and activity [106,107,108]. This suggests that TF inhibition serves as a therapeutic target for suppressing abnormal platelet activation, thereby improving renal function, and clearing IS may effectively alleviate platelet disorders in patients with CKD.

#### 3.2.2. DAMPs and PAMPs

To perform their immunological and inflammatory functions, platelets express pattern recognition receptors (PRRs), including Toll-like receptors (TLRs), nucleotide oligomerization domain-like receptors (NLRs), and C-type lectin receptors, which play vital roles in sensing and responding to PAMPs or DAMPs [25,109]. PAMPs are molecules derived from microorganisms and typically represented by LPS [110]. In contrast, DAMPs are released from stressed or dying cells [110]. Except for the DAMPs produced by the kidney, DAMPs and PAMPs originating from remote sites of injury or infection can also enter the nephron through circulation [111].

LPS, as a type of PAMP, has been reported to cause platelet activation and accumulation in the lungs of septic mice [112,113]. In AKI, LPS can significantly increase the expression of TLR-4, P-selectin, and integrin α_IIb_β_3_ in platelets, and these effects were mitigated by a TLR4 inhibitor, effectively preventing kidney and lung injury, as well as inflammatory cell infiltration into the kidney and lung tissue [114]. TLRs can also recognize endogenous ligands released during tissue or cell injury. When activated, TLRs trigger intracellular signals and ultimately synthesize inflammatory molecules through the nuclear factor kappa-light-chain-enhancer of activated B cells or activator protein 1 pathway [115]. In AKI models, the levels of urinary mitochondrial DNA, inflammation markers, and the platelet activation marker platelet factor 4 (PF4) significantly increased and positively correlated with renal injury and inflammation in vivo. Patients with AKI showed the same changes [116,117]. Moreover, platelet microthrombus formation has been observed in the corticomedullary region of the kidney in AKI mice [117]. Additionally, it has been confirmed in vitro that mitochondrial DNA (mtDNA) stimulates platelet activation [116]. Other DAMPs, such as high-mobility group box 1 (HMGB1), are currently gaining more attention. HMGB1 is upregulated in various renal diseases, including AKI, CKD, primary glomerulonephritis, nephrotic syndrome, secondary renal diseases (anti-neutrophil cytoplasmic autoantibody-associated vasculitis, lupus nephritis (LN), diabetic nephropathy, and hemolytic uremic syndrome), nephrolithiasis, and renal carcinoma [118]. Some studies have shown that HMGB1 causes platelet activation through TLR4 [119]. Therefore, it is intriguing to note the possible existence of a loop between platelets and DAMPs, where DAMPs can activate platelets, which can subsequently release DAMPs [116,119,120], thereby enhancing the progression of kidney disease, as discussed later.

Recent studies have demonstrated that DAMPs can cause platelet pyroptosis. This programmed cell death induces thrombocytopenia and the release of inflammatory cytokines, such as IL-1β and IL-18, promoting platelet aggregation, vaso-occlusion, endothelial permeability, and cascaded inflammatory response [121]. Since urate crystals, ROS, extracellular ATP, and nucleic acids are common DAMPs that can be recognized by the NLR thermal protein domain associated protein 3 in kidney diseases [115], it is highly likely that platelets also undergo severe pyroptosis in kidney disease, leading to microcirculation and inflammatory disorders and disease progression. However, to date, no relevant research has been conducted on this topic.

Overall, platelets participate in the inflammatory reactions and immune processes in kidney diseases. Therefore, control over PAMPs/DAMPs is the key to suppressing platelet abnormalities during kidney disease.

#### 3.2.3. Uremic Toxin Accumulation

Uremic toxins accumulate due to the deterioration of kidney function. Therefore, the accumulation of abnormal components in the body should have an impact on platelets, which is supported by the following two points. First, some omics studies indicate that the transcriptome and proteomics of platelets in patients with CKD have indeed changed, and the changes in those with ESRD are more significant; however, dialysis can reverse some changes [13]. Second, consistent with this finding, it has been clinically observed that renal replacement therapy can alleviate uremic bleeding. However, the results of studies on the effects of urinary toxins on platelets are inconsistent.

The polyamines spermidine, spermine, and putrescine have been shown to impair platelet responsiveness as indicated by platelet aggregation, secretion, and thromboxane synthesis [122]. Other toxins that dialysis can clear, such as phenol, phenolic acids, and guanidinosuccinic acid, also affect platelet function. Phenolic acids impair the primary aggregation of platelets. Guanidinosuccinic acid promotes NO synthesis and prevents the second wave of ADP-induced platelet aggregation [10]. These findings partially explain the alleviation of bleeding after renal replacement therapy. Additionally, a functional defect in the von Willebrand factor–platelet interaction may contribute to abnormal hemostasis, which further affects the function of the integrin α_IIb_β_3_ complex [10]. Overall, uremic toxins increase the risk of bleeding in patients with renal dysfunction; however, the specific underlying mechanisms remain to be fully elucidated, requiring further investigation. Conversely, some studies have suggested that uremic toxins can alter platelet function and promote thrombosis. IS contributes to CKD-associated thrombosis by inducing platelet hyperactivity, including an elevated response to collagen and thrombin, an increase in platelet-derived microparticles, and platelet–monocyte aggregation [108]. Moreover, IS has been demonstrated to increase the expression of P-selectin and integrin α_IIb_β_3_ in a dose-dependent manner, and activate the collagen pathway [108,123]. Another possibility is that platelet fibrinogen receptors may exhibit an impaired ability to undergo conformational changes and bind to the ligand under uremia condition [124], while dialysis removes these influencing factors, allowing them to exhibit their original procoagulant state.

Overall, patients with renal failure not only face a high risk of bleeding but also have thrombotic tendencies, which cannot be attributed to a single abnormal factor and frequently leave nephrologists perplexed. The mechanisms underlying the regulation of platelet function by uremic toxins are complicated, and remain elusive; therefore, further observational studies based on a larger and less heterogeneous population and delicate mechanistic studies are needed. Other conditions accompanying renal failure, including hyperparathyroidism and hyperlipidemia, may also impact platelet function [10,125] and are not easily corrected by renal replacement therapy.

### 3.3. Anemia and Hemodynamic Changes

Anemia is a common complication in patients with renal disease. Significant anemia has been associated with an increased risk of bleeding, and red blood cell count is negatively correlated with bleeding time [126]. Notably, red blood cells in the bloodstream not only alter the hemodynamic state, but also release substances that directly affect platelet function. By driving platelets away from the axial flow and toward the vessel wall during normal laminar flow, erythrocytes in the vessel’s center promote platelet-vessel wall interactions [127]. Additionally, they enhance platelet function by generating ADP [128] and thromboxaneA_2_ (TXA_2_) [129], inactivating PGI2 [130], and scavenging NO [131]. Accordingly, correcting anemia appears to lower the risk of bleeding, while stimulating hematopoiesis using erythropoietin increases platelet reactivity and, correspondingly, increases the occurrence of ischemic events [132,133,134]. Nonetheless, compared to traditional treatments, roxadustat and other hypoxia-inducible factor-prolyl hydroxylase inhibitors have recently been approved for treating chronic renal anemia, and are associated with a relatively low risk of cardiovascular events compared to traditional treatments [135,136,137]. Furthermore, they can ameliorate systemic inflammation and potentially benefit iron and lipid metabolism [138,139,140]. Therefore, compared to traditional treatments, hypoxia-inducible factor-prolyl hydroxylase inhibitors may benefit platelet function in patients with renal failure. Thus, further studies are required to evaluate the important aspects of hypoxia-inducible factor-prolyl hydroxylase inhibitors, such as appropriate hemoglobin targets, and the risk of thrombosis and bleeding.

Although the cardiac output may remain unchanged, almost all kidney diseases are affected by changes in renal hemodynamics. Since hemodynamic changes occur quickly, renal hemodynamic dysregulation is an essential hallmark of AKI [141]. Hemodynamic changes in CKD are relatively slow. Both of these conditions lead to hypoxic regions in the kidney [142,143], and hypoxia induces endothelial activation, followed by leukocyte stasis and blocking of blood flow to peritubular capillaries, ultimately leading to the loss of the capillary network and promotion of inflammation and changes in platelet status. Furthermore, certain conditions, including hyperglycemia, can not only lead to glycosylation of the endothelial system in the kidney, but can also systemically increase blood viscosity and cause an osmotic impact on platelets, resulting in enhanced platelet reactivity and increased interaction with the endothelium [144].

### 3.4. Other Influencing Factors

Renal disease associated with lupus, hemolytic uremic syndrome, or thrombotic thrombocytopenic purpura may be accompanied by a significant reduction in platelet count. In addition to the above mechanisms, the main reason is the abnormal activation of antibodies and complements, which directly leads to platelet destruction or coagulation dysfunction, resulting in excessive platelet consumption [145,146]. Heparin (used for dialysis anticoagulation) may activate platelets and induce thrombocytopenia via immunologic mechanism [147]. Complement activation and substantial thrombocytopenia frequently occur during dialysis due to the interaction of blood with relatively bioincompatible membranes [10]. Moreover, patients with uremia use antibiotics, such as β-lactam antibiotics, that could raise the risk of hemorrhage by interfering with ADP receptors and impairing platelet membrane function [10].

The kidney is highly susceptible to hypoxia, which is frequently associated with kidney disease progression. Thus, hypoxia plays a critical role in exacerbating oxidative stress in CKD [148]. Recent studies have revealed that ROS play a crucial role in regulating platelet activation, aggregation, and recruitment, with the ability to trigger platelet apoptosis. Therefore, exogenous ROS produced by other cells during kidney disease can substantially affect platelet function [149]. A previous study provided evidence supporting this perspective, showing that glomerular ROS production increased in rats with diabetic nephropathy. Treatment with the antiplatelet drug sarpogrelate improved the ROS/NO imbalance in glomeruli, inhibited platelet aggregation, reduced the levels of microparticles derived from platelets, and lowered proteinuria levels [150].

Overall, kidney disease involves complex pathological and physiological changes; thus, the mechanism of platelet abnormalities is inevitably intricate. Additionally, understanding how kidney disease affects platelets and the key contributing factors may provide insights into treating kidney disease and managing platelet abnormalities.

## 4. Platelet in Inducing Progression of Renal Disease

**Figure 4:**
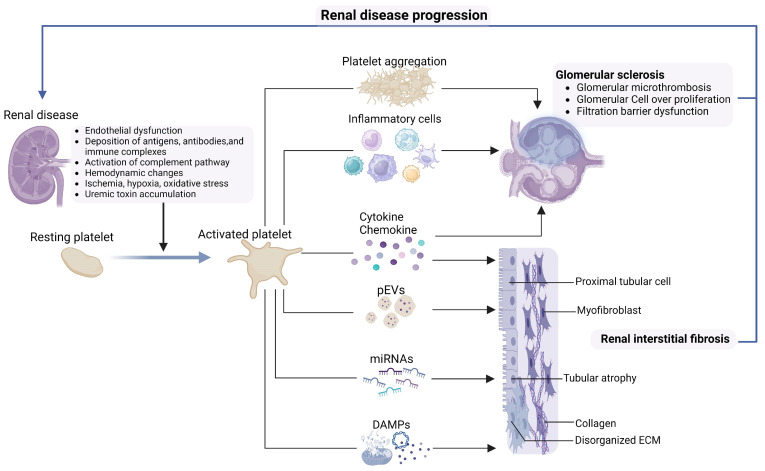
The interaction between renal disease and platelets. The various pathological and physiological processes of kidney disease provide conditions for promoting platelet activation. Activated platelets induce glomerular thrombosis, leading to nephron ischemia and hypoxia. Platelets release various cytokines and chemokines, recruit inflammatory cells, and promote the destruction of glomerulus and extracellular matrix (ECM) disorganization in tubulointerstitium, ultimately leading to glomerulosclerosis and tubulointerstitial fibrosis. In addition, miRNAs and DAMPs released by activated platelets can directly cause or promote renal fibrosis. Platelet-derived extracellular vesicles (pEVs) can migrate to tissue structures that platelets cannot reach, and their cargos may also cause alteration in renal cells. Therefore, platelets promote the progression of kidney disease, which in turn provides more conditions for platelet activation. Created with BioRender.com.

The above discussion summarizes the typical mechanisms underlying platelet abnormalities in kidney diseases. However, the relationship between kidneys and platelets is not unidirectional (Figure 4). Platelets are found in glomerular structures in glomerulonephritis and even in some animal models of nephritis, where platelet protein levels in plasma and urine correlate with proteinuria and renal histological abnormalities [151]. Since platelets participate in various physiological processes involving hemostasis, immunity, and inflammation, they can also participate in the pathogenesis of kidney disease through various mechanisms [152,153]. During kidney disease, platelets are activated, and their secretions are localized within glomerular structures. The pathogenesis by which platelets may participate in the progression of kidney disease is as follows: first, platelets interact with damaged or activated renal endothelium to form microthrombosis, leading to glomerular microcirculation disorders, promoting ischemia and hypoxia; second, the interaction between platelets and leukocytes promotes inflammation and immune processes, releases inflammatory mediators, increases glomerular permeability, and damages glomerular selective filtration function. Moreover, activated platelets have active secretory functions, and their different secretory products (such as growth factors and DAMPs) directly or indirectly promote glomerular remodeling and sclerosis.

### 4.1. Glomerular Microthrombus

Platelet aggregation and thrombosis in the glomerular capillaries and small arteries within the kidney are typical renal pathological features of systemic lupus erythematosus (SLE), thrombotic thrombocytopenic purpura, hemolytic uremic syndrome, systemic sclerosis, and acute and chronic transplant rejection [154,155,156,157]. Additionally, platelet aggregation may be observed within the glomerulus in some forms of glomerulonephritis, including mesangiocapillary glomerulonephritis and immunoglobulin A (IgA) nephropathy [158,159,160]. In addition to the complement system and antibodies participating in platelet hyperactivation, the main cause of glomerular microthrombus formation is the disruption of endothelial integrity, with activated platelets playing a role in hemostasis and vascular repair following injury [161]. In mouse models of glomerulonephritis, rapid platelet aggregation was observed after the injection of anti-glomerular basement membrane antibodies, which was primarily mediated by the binding of the platelet surface receptor GPVI to collagen. This not only suggests that platelet aggregation can occur independently of GPIb-V-IX complexes, but also indicates that platelets can be rapidly recruited to form microthrombi in response to glomerular injury, contributing to disease progression [162], and even platelet abnormalities may occur earlier than other changes. Therefore, early targeted platelet interventions may effectively inhibit kidney disease progression. Studies have demonstrated that in rat models of diabetic nephropathy, increased levels of urinary TXB_2_ (a biomarker for TXA_2_ in vivo) correlate with elevated proteinuria and characteristic histopathological features, including glomerular thrombosis and sclerosis. The administration of TXA_2_ synthase inhibitors decreased glomerular thrombus formation and effectively improved proteinuria [163]. Additionally, TXA_2_ has been implicated in both inflammatory [164,165] and non-inflammatory [166] kidney conditions. In these diseases, the urinary levels of TXA_2_ are elevated, which can amplify the formation of renal immune complexes and exacerbate renal inflammation. TXA_2_ synthase inhibitors have been demonstrated to significantly improve renal function and slow the course of renal diseases. Collectively, these findings suggest that TXA_2_ serves as a key intermediary in renal injury progression across various kidney diseases [167]. Renal histopathological findings objectively indicate that platelets participate in kidney disease progression. Furthermore, interventions targeting platelets in animal models of kidney disease have demonstrated that platelets not only participate in the progression of kidney disease, but also play an important role in driving the development of kidney disease.

Despite being an organ with an abundant blood supply and average of a million nephron per kidney, the kidney is highly susceptible to hypoxia due to its unique structure (oxygen shunt diffusion between the arterial and venous vessels). Hypoxia plays a significant role in the development and progression of kidney diseases. Basic and clinical studies have established that hypoxia-induced renal fibrosis is a common pathological hallmark of CKD, and that renal hypoxia is associated not only with CKD, but also with the AKI-to-CKD transition [148]. Renal hypoxia is both a cause and a result of kidney disease progression, leading to a vicious cycle of worsening kidney disease [148]. Platelet thrombi can cause microvascular obstruction, resulting in ischemia and hypoxia, which promotes the abovementioned vicious cycle. Importantly, this cycle is not limited to the glomerular microcirculation. Sclerosis of the “parent” glomeruli can further lead to stasis of the peritubular capillaries, resulting in tubulointerstitial hypoxia and fibrosis [148]. Furthermore, activated platelets can generate additional ROS, which may exacerbate oxidative stress in hypoxic kidneys and tissue damage [149]. Microvascular impairment arising from microthrombi formed by platelets could magnify “danger signals”, resulting in the involvement of the surrounding tissues.

The formation of microthrombi results in widespread and severe renal hypoxia, which promotes renal disease progression. Moreover, platelet thrombi are not simply static plugs. Activated platelets can recruit leukocytes, secrete cytokines, and promote inflammation and immune responses (discussed later), further exacerbating kidney injury.

### 4.2. Platelets Promote Renal Inflammation and Fibrosis

Chronic detrimental stimuli can induce dysregulation of the normal repair process, the persistence of the inflammatory response, and the accumulation of extracellular matrix (ECM), resulting in renal fibrosis [168]. Renal fibrosis is the ultimate pathological alteration in progressive kidney disease, and is closely associated with reduced kidney function. During this process, beyond their basic hemostatic function, platelets also facilitate inflammation by mediating leukocyte chemotaxis and adhesion, which subsequently contributes to renal fibrosis.

#### 4.2.1. CD40/CD40L(CD154)

Activated platelets express CD40 and CD154; CD154 is also known as CD40L. Both platelets and endothelial cells express the transmembrane glycoprotein receptor CD40. Notably, CD40L in platelets is consistently present in a soluble form, and is only expressed upon activation [169]. Consequently, platelets are the principal source of soluble CD40L [170]. Podocytes in human glomeruli can also express CD40 [171]. In response to platelet CD40L and activated platelet supernatant, podocytes release matrix metalloproteinase-9. This effect can be mitigated by anti-CD40 antibodies, suggesting that activated platelets influence podocyte function and basement membrane remodeling during glomerular inflammation [171]. A cohort study involving 243 patients with CKD has revealed that circulating soluble CD40L levels are associated with changes in kidney function in these individuals [172]. Consistent with this finding, another descriptive study showed elevated platelet microparticles and significantly increased CD40L levels in patients with CKD [173]. Moreover, a prospective study on DKD demonstrated that plasma soluble CD40L levels were higher in patients with DKD than in those with normal albuminuria [174]. It has been suggested that increased CD40L in circulation can be a risk factor for early kidney disease development in patients with type 1 diabetes [175]. Additionally, CD40L can bind to CD40 receptors on B cells, induce B cell proliferation, promote B cell differentiation, block B cell apoptosis, and mediate antibody class switching. Activated platelets can enhance lymphocyte-endothelial cell adhesion, induce T-cell reactions, and migrate toward inflammatory regions. In kidney diseases, platelet CD40L may serve as a link between innate and adaptive immunity [176].

#### 4.2.2. Adhesion Molecules

Adhesion molecules are a group of molecules comprising various ligand/receptor molecules that promote cell-to-cell and cell-to-ECM adhesion [177]. Adhesion molecules are essential in both healthy and diseased states for processes including white blood cell circulation and movement, cell differentiation, tissue maintenance, and immune cell activation and communication [178]. The expression of adhesion molecules in the tissues is significantly elevated when the tissues are affected by either acute or chronic inflammatory disorders. The expression of adhesion molecules is higher in platelets from people with type 2 diabetes mellitus [144]. Renal diseases that involve kidney IRI, tubulointerstitial nephritis, and allograft rejection are also associated with adhesion molecule-mediated leukocyte binding. In the glomeruli and interstitium, leukocytes (mainly monocytes/macrophages) aggregate, and their infiltration is mediated by adhesion molecules [179].

Upon platelet stimulation, P-selectin (CD62P), which is a marker of platelet activation, is rapidly redistributed from α-granules to the platelet surface [180]. It promotes platelet and endothelial cell adhesion to leukocytes, chemotaxis, leukocyte adhesion, and migration by interacting with P-selectin glycoprotein ligand-1 on the surfaces of monocytes and neutrophils. Additionally, it promotes the maturation of active monocytes into macrophages, which play an essential role in inflammation [181]. In non-pathological conditions, renal endothelial cells do not express P-selectin; however, research has shown that P-selectin expression is increased in renal diseases, such as diabetic nephropathy and glomerular diseases (IgA nephropathy, minimal change disease, membranous nephropathy, mesangial proliferative glomerulonephritis, and LN) [182,183], particularly in diabetes when circulating P-selectin levels are significantly elevated, and even higher in diabetes with nephropathy [180,182]. Although the specific source of P-selectin under these conditions is not entirely clear, it has been confirmed that P-selectin initiates leukocyte migration and accumulation in the renal glomeruli and promotes renal inflammation by mediating endothelial cell–leukocyte binding. Studies on cell adhesion and signaling mechanisms dependent on selectins can further elucidate the mechanisms of inflammatory immune responses and disease development, and provide new approaches for clinical disease treatment by developing and improving specific monoclonal antibodies or ligand antagonists targeting the functional sites of P-selectin.

Thrombospondin-1 is also an adhesion molecule with a role in kidney function. In the kidney, thrombospondin-1 is the main activator of transforming growth factor beta (TGF-β), which is a central driver of renal fibrosis in renal cells, and pro-inflammatory effects have been documented in both in vitro and in vivo settings [184]. Researchers have found a correlation between thrombospondin-1 levels and the severity of renal injury and vascular lesions in patients. This is related to its ability to enhance the bioactivity of TGF-β, mediating glomerular injury response through glomerular hypertrophy, matrix expansion, and glomerulosclerosis [184]. Based on these findings, elevated adhesion molecule levels in the peripheral blood may indicate vascular endothelial cell damage and leukocyte activation in patients with renal disease.

#### 4.2.3. Chemokine

PF4 (CXCL4) is a heparin-binding protein that makes about 2% of platelet α-granules and is secreted in a P-selectin-dependent manner. PF4 interacts with many cells and has a strong chemotactic effect on neutrophils, monocytes, and fibroblasts [185,186]. In addition to facilitating neutrophil activation and adherence to endothelial cells, PF4 promotes phagocytosis in monocytes, as well as the chemotaxis of T cells [186]. Many studies on PF4 have focused on heparin-induced thrombocytopenia, and research has suggested that the formation of IgG specific to PF4-H antibodies is associated with increased mortality rates in patients undergoing hemodialysis [187]. Moreover, studies have suggested that PF4 levels are elevated in DKD, with PF4 levels in insulin-dependent patients with diabetes with substantial albuminuria being significantly elevated compared to those in patients with diabetes with mild albuminuria and healthy controls [188]. Several prospective clinical studies on SLE have indicated that the urine concentration of PF4 is considerably higher in patients with active LN than in those with active non-renal SLE, non-active SLE, or healthy controls, indicating that PF4 is a potential non-invasive biomarker for predicting kidney disease activity in SLE [189,190,191]. However, a previous study has shown that PF4 can alleviate kidney transplant rejection in mice. On the 56th day post-transplantation, PF4 significantly ameliorated vascular and glomerular alterations, interstitial inflammation, fibrosis, and tubular atrophy. This effect may be related to the ability of PF4 to reduce the production of IL-17 in vivo and to limit T helper 17 cells in vitro [192]. Currently, the mechanism of PF4 function in kidney disease remains poorly understood; thus, further research is needed to clarify this.

β-Thromboglobulin (β-TG), which is another chemokine, is a PF4 and platelet base protein cleavage product that accounts for 10% of platelet α-granules [193,194,195]. Structurally, it is 50% similar to PF4 and is excreted in the urine [195]. Previous studies have indicated that plasma β-TG and PF4 levels are significantly higher in patients with chronic renal failure than in healthy individuals, and their elevated levels are negatively correlated with blood urea nitrogen, creatinine, and creatinine clearance [196,197]; however, in patients with DKD, urinary β-TG levels are considerably higher than in healthy controls. Urinary excretion of β-TG strongly correlates with blood creatinine and β-microglobulin concentrations [198]. These findings suggest a correlation between the urinary excretion of β-TG and glomerular filtration indicators, making it a potential clinical indicator of glomerular filtration function.

#### 4.2.4. Protease-Activated Receptors (PARs) and Platelet-Activating Factor (PAF)

PARs are members of the transmembrane G protein-coupled receptor family. PAR1 and PAR4 can be detected in human platelets, and thrombin activates platelets by cleaving PARs [199,200]. PARs have been shown to play a role in AKI and CKD [201]. For example, Lok et al. [202] demonstrated that the expression of PAR1 in renal tubules increased in the experimental renal fibrosis model of unilateral ureteral obstruction and the AKI-CKD transition model of unilateral IRI, whereas the use of PAR1 antagonists significantly reduced kidney injury and fibrosis in both models. Mechanistically, inhibiting PAR1 can suppress mitogen-activated protein kinase extracellular signal-regulated kinase 1/2 and TGF-β-mediated Smad signaling and inhibit excessive oxidative stress, pro-inflammatory cytokine expression, and macrophage infiltration into the kidney. Moreover, in crescentic glomerulonephritis, PAR1 mediates thrombin-dependent cell-mediated renal inflammation [203]. Although the source of platelet-derived PAR-1 is unclear, this suggests that activated platelets promote renal fibrosis through this pathway, and targeting PAR1 inhibition is an effective method to treat renal fibrosis.

PAF is a pro-inflammatory lipid mediator mainly produced by platelets with various biological effects related to glomerular injury. During the renal inflammatory process, PAF may exert detrimental effects via chemoattraction, activating leukocytes, activating the complement system, contraction, and stimulating mesangial cells to produce leukotrienes and oxygen free radicals [204,205]. When PAF binds to receptors, it has biological actions, such as inducing macrophages to secrete IL-1, IL-6, and tumor necrosis factor-alpha and stimulating B lymphocytes to produce IgG and IgE [181]. In a study, PAF infusion caused a significant increase in urinary protein excretion, which was reversible after stopping the infusion, suggesting that PAF alters glomerular selective filtration [206]. In an obstructive kidney disease model, PAF receptor signals promoted the formation of a pro-inflammatory environment by regulating collagen deposition and the ECM, which is conducive to the fibrosis process [205]. Similarly, in the DKD model, PAF can stimulate the deposition of ECM in mesangial cells by activating the protein kinase C-TGF-β1 axis, increasing the risk of glomerular fibrosis. Additionally, PAF production increased in the glomeruli in other types of glomerular injury, such as nephrotoxic nephritis, proliferative glomerulonephritis, and LN, and the use of PAF receptor antagonists could improve lesions [10,207]. In a mouse LN model, PAF levels in the plasma and urine were related to proteinuria and renal histological abnormalities [151], and the treatment with PAF receptor antagonists alleviated proteinuria and improved survival [207].

In addition to the factors above derived from platelets involved in renal inflammation and fibrosis, recent studies have revealed that interactions between platelets and leukocytes can promote the formation of neutrophil extracellular traps (NETs), which may also facilitate kidney disease progression. In a mouse model of I/R-induced AKI, Jansen et al. demonstrated that clopidogrel could reduce renal NETs formation, reducing tissue injury and preserving kidney function [208]. Moreover, NETs formation is associated with the progression of several kidney diseases [209,210]. Mechanically, NETs can be induced by platelets in various approaches. The interaction between platelet integrin α_IIb_β_3_ and neutrophil CD11b can promote NETs formation in the presence of fibrinogen. Additionally, TLR4 can mediate the interaction between platelets and neutrophils to facilitate NETs formation [144]. In addition to regulating NETs formation, platelets have also been demonstrated to regulate the formation of macrophage extracellular traps. Using the AKI model induced by rhabdomyolysis, Okubo et al. demonstrated that heme-activated platelets augmented the formation of macrophage extracellular traps by boosting intracellular ROS and histone citrullination [211]. Whether platelets promote macrophage extracellular trap formation in other kidney diseases remains unclear. However, the mentioned mechanisms may elucidate methods of kidney disease treatment.

### 4.3. Other Components Derived from Platelets

In addition to the aforementioned mechanisms, platelets promote kidney disease progression by releasing other mediators. Platelet-derived extracellular vesicles (pEVs) release some of these mediators. Platelets are prone to generating pEVs when stimulated by various activators, and these vesicles were initially identified as procoagulant particles. However, beyond hemostasis, the cargo of pEVs is remarkably diverse and encompasses lipids, proteins, nucleic acids, and organelles that participate in various biological processes [212,213]. According to research, pEVs may amplify inflammation by activating leukocytes and endothelial cells via the surface markers CD41 and CD62P [214]. Moreover, the potential of pEVs to traverse tissue barriers enables the delivery of platelet-derived content to cellular recipients and organs that are inaccessible to platelets [213]. Previous studies have identified the kidney as a primary target organ for pEVs [212], which are likely involved in the crosstalk between the kidney and other organs and between various cell types within the kidney. Although the mechanisms of pEVs in kidney disease are not fully known, they could have significant implications for kidney disease progression.

#### 4.3.1. Platelet-Derived DAMPs

Upon stimulation, platelets expressing Fcγ receptor (FcγR) IIA release pEVs, which interact with circulating neutrophils, ultimately depositing and forming thrombi in several organs including the kidneys [215]. Subsequent research has shown that immune complexes deposited in the kidneys can stimulate the activation of platelet FcγRIIA, leading to the further release of mitochondria, either in free form or embedded within pEVs, which act as self-antigens and DAMPs, thus promoting immune responses [18]. This suggests that platelet-mediated adaptive immunity plays a role in the development of immune-related kidney diseases, and that this mechanism is likely applicable to many other immune-mediated inflammatory disorders, as IgG-containing immune complexes have been identified in various diseases, including systemic sclerosis, rheumatoid arthritis, vasculitis, and Sjogren’s syndrome [216].

HMGB1 is an important nonspecific inflammatory mediator that when activated promotes NETs formation [217,218] and the release from platelets, which enter the endosomal system through receptor for advanced glycation endproducts (RAGE)-mediated internalization of the complex. The release of NETs can continuously activate platelets, which subsequently trigger a series of coagulation factors, including factor XII and thrombin, thereby accelerating the activation of the extrinsic coagulation pathway and promoting microthrombus formation [219]. Additionally, the expression of HMGB1 can increase the recruitment of monocytes, and the oxidation and release of active HMGB1 can cause platelet aggregation, further activating monocyte TLR2 through RAGE, leading to the local release of TF and inflammatory cytokines [220]. TLR2 and TLR4 deficient mice in DKD mouse models showed reduced myeloid differentiation primary response 88 signaling in the kidney and diminished renal inflammatory damage [221]. Similarly, RAGE-deficient mice showed delayed progression of DKD [222]. Therefore, TLR2, TLR4, and RAGE activation are the mechanisms by which DAMPs promote immune-mediated injury, and the removal or neutralization of extracellular HMGB1 can improve renal damage. Targeting this pathway has great potential for diagnosing and treating glomerular microthrombosis in DKD.

In addition to classic DAMPs, a recent study using proteomic techniques has discovered that activated platelets release transthyretin, which may also serve as a DAMP that promotes sepsis-induced AKI [223].

#### 4.3.2. Growth Factor and Chemokine

Activated platelets release mediators that directly affect renal glomerular cell function and promote renal fibrosis. The platelet-derived growth factor (PDGF)/PDGF receptor signaling pathway is one of the most extensively studied in the field of progressive kidney disease, and has been confirmed to regulate various pathophysiological activities, including cell proliferation, migration, survival, and ECM deposition, promoting renal fibrosis [224,225,226]. Additionally, TGF-β, which is recognized as a key factor in fostering renal fibrosis, can be released by platelets. Several studies have demonstrated that platelet counts positively correlate with the concentration of TGF-β in peripheral blood, confirming their important role as TGF-β activity carriers [227]. Furthermore, C-C motif chemokine ligand (CCL)5 or regulated upon activation, normal T cell expressed and presumably secreted (RANTES) is a chemokine stored in platelet granules, which is a chemoattractant for eosinophils, monocytes, and T lymphocytes [228]. RANTES released as pEVs can be deposited into damaged or activated endothelial cells, enhance monocyte recruitment, and regulate leukocyte adhesion and rolling [186]. A study revealed that RANTES expression was elevated in the renal tissues of patients with diabetic nephropathy and that urinary RANTES was correlated with proteinuria levels, declining kidney function, and interstitial fibrosis in these patients, suggesting that RANTES contributed to the development of DKD by recruiting and activating macrophages/monocytes and lymphocytes. Urinary RANTES levels may be potential prognostic biomarkers for diabetic nephropathy [229,230].

In addition to its antiplatelet activation effect, targeting several potent profibrotic factors such as PDGF and TGF-β may prevent organ fibrosis without affecting other platelet functions.

#### 4.3.3. MicroRNAs (miRNAs)

Platelets lack nuclei or genomic DNA; however, patients with CKD displayed altered platelet mRNA and miRNA transcriptomes compared to controls, and partially restored after dialysis [13]. miRNAs derived from circulating activated platelets or platelet microparticles may be promising non-invasive biomarkers for patients with CKD, according to current research [231]. Among these, miRNA-21 found across all cell types, with platelets being the main source, plays a clear role in promoting renal fibrosis in mice [232]. Eliminating miRNA-21 in mice significantly reduced interstitial fibrosis, glomerulosclerosis, tubular injury, and inflammation and halted CKD progression. The inhibition of microRNA-21 protects glomerular and interstitial cells against fibrosis and inflammation induced by TGF-β. Mechanically, this could result from improved mitochondrial function and enhanced peroxisome proliferator-activated receptor/retinoid X receptor activity [231]. Through the extracellular signal-regulated kinase 1/2 and TGF-β/Smad signaling pathways, miRNA-21 is also involved in diabetic models of renal fibrosis [231]. Thus, the miRNA-21 is a potential antifibrotic therapy target. Additionally, miRNA-223, which is the most prevalent miRNA in platelet microvesicles, can migrate to vascular endothelial cells and is associated with various inflammatory diseases. Previous studies have shown that miRNA-223 is closely associated with NLR thermal protein domain-associated protein 3 genes in IgA nephropathy. Therefore, we have reason to believe that miRNA-223 may promote renal fibrosis by activating inflammation [231]. Furthermore, research has demonstrated that miRNA-124 in an IgA nephropathy model can activate profibrotic genes in podocytes and tubular cells [231]. Notably, studies have shown that miRNA-24, miRNA-191, miRNA-126, miRNA-150, and miRNA-197 can also be released into the circulation in the form of pEVs, and serve as potential biological markers for CKD [231], whereas research in this area is still limited.

With the advancement of molecular biology technologies, more studies have focused on the functions of miRNAs. However, the field of kidney disease requires further investigation of platelet-derived miRNAs. Platelet-derived miRNAs play a remarkable role in regulating fibrosis in CKD; targeting specific miRNAs may be an exceptional therapeutic strategy for renal fibrosis. Therefore, more intensive research is needed to identify the biological mechanisms through which miRNAs contribute to renal disease progression.

## 5. Conclusions and Perspectives

Abnormal platelet function frequently occurs in patients with renal disease, particularly those with CKD, who usually experience two entirely different and paradoxical hemostatic events. This dilemma has been well described elsewhere (reviewed in refs. [122,124]); however, the mechanisms underlying platelet dysfunction in renal disease remain unclear.

In this study, we attempted to clarify the association between platelet dysfunction and kidney disease progression. A vicious cycle exists between platelet dysfunction and disease progression in most kidney disease cases. Kidney disease creates a disordered environment that leads to platelet activation. Additionally, activated platelets promote the progression of kidney disease through the formation of microthrombi and the mediation of inflammation and immune responses. Therefore, identifying the key links that can break this cycle is crucial. However, conducting such a review is challenging. First, most studies linking kidney disease to platelets are observational, and no uniform method exists for platelet function testing. Furthermore, most clinical studies cannot include meticulous grouping based on etiology, renal function grading, complications, and treatment measures. Consequently, data inconsistencies hamper the generation of compelling conclusions. Moreover, most mechanistic experiments on this topic are animal-based, and there are limited investigations of the relationship between primary glomerular diseases and platelets. Previous studies were predominantly focused on AKI and DKD. This lack of research has hindered our understanding of the fundamental interactions between the kidneys and platelets.

Therefore, by elucidating the specific relationship between platelets and renal disease, clinicians can precisely intervene in platelet hemostasis, inflammation, and immune processes, thereby avoiding the risk of bleeding caused by the global inhibition of platelet activation. Achieving this goal undoubtedly requires extensive experimentation. In the future, various omics-based approaches, including proteomics and transcriptomics, could be used to investigate changes in platelet function in different types and stages of renal disease. Additionally, specific knockout of platelets or platelet-related molecules could help to clarify the precise contribution of platelets to kidney disease.

## Figures and Tables

**Table 1:** Overview of contents of platelet granules [19].

Granule	Type	Contents
α-granules	Adhesive proteins	P-selectin, Von Willebrand factor, Fibronectin, Vitronectin, Fibrinogen
	Integral membrane proteins	Integrin αIIbβ3, GPIba-IX-V, GPVI, TLT-1
	Chemokines	CXCL1, CXCL2, CXCL5, CXCL6, CXCL7, CXCL8 (IL 8), CXCL-12, CCL2, CCL3, CCL5 (RANTES), CCL7, IL1β, CD40L Proteases
	Growth factors	Transforming growth factor β (TGF-β), Platelet-derived growth factor (PDGF), Vascular endothelium growth factor (VEGF), Fibroblast growth factor (FGF), Insulin-like growth factor 1 (IGF-1), Epidermal growth factor (EGF)
	Coagulation factors	Factor V, Protein S, Factor XI, Factor XIII
	Immune mediators	Complement C3 precursor, Complement C4 precursor, Factor D, Factor H, C1 inhibitor, Immunoglobulins
δ-granules	Nucleotides	ATP, ADP, GTP, GDP
	Bivalent cations	Ca^2+^, Mg^2+^
	Amines	Serotonin, Histamine
Lysosome	Glycohydrolases	Heparinase, β-N-acetyl-glucosaminidase
	Acid proteases	Aryl sulphatase, Cathepsins, Collagenase, Acid phosphatase

TLT-1, TREM-like transcript 1; CXCL, C-X-C motif chemokine ligand; IL, interleukin; CCL, C-C motif chemokine ligand.
